# Comparing the efficacy and safety of direct oral anticoagulants with vitamin K antagonists in dialysis patients with nonvalvular atrial fibrillation: a systematic review and meta-analysis

**DOI:** 10.1007/s00392-025-02711-7

**Published:** 2025-07-28

**Authors:** Tian Li, Tong Li, Yujun Xu, Diona Gjermeni, Lukas Heger, Dirk Westermann, Christoph B. Olivier

**Affiliations:** 1 Department of Cardiology and Angiology, University Heart Center Freiburg – Bad Krozingen, Faculty of Medicine, University of Freiburg, Hugstetter Str. 55, 79106 Freiburg, Germany; 2 Department of Cardiac Surgery, Heinrich-Heine-University Medical School, Duesseldorf, Germany; 3 Faculty of Medicine, Institute for Medical Information Processing, Biometry and Epidemiology (IBE), LMU Munich, Munich, Germany

**Keywords:** Chronic kidney failure, Dialysis, Atrial fibrillation, Anticoagulation, Direct oral anticoagulant, Vitamin K antagonist

## Abstract

**Aims:**

For patients with atrial fibrillation (AF) and preserved renal function, direct oral anticoagulants (DOACs) are superior to vitamin K antagonists (VKAs) for stroke prevention. However, the evidence in patients with end-stage kidney disease (ESKD) on dialysis remains inconclusive. In this systematic review and meta-analysis, we aim to compare the efficacy and safety of DOACs and VKAs in dialysis patients with nonvalvular AF.

**Methods and results:**

We conducted a systematic literature review of publications comparing DOACs and VKAs in dialysis patients with nonvalvular AF. Data of RCTs and cohort studies were synthesized separately. Outcomes were reported as risk ratios with 95% confidence intervals. Heterogeneity was assessed using *I*^2^ statistics.

Ten studies were included in this meta-analysis: 4 RCTs (DOACs, 269 patients; VKAs, 217) and 6 cohort studies (DOACs, 7039 patients; VKAs, 22,983). In RCTs, the risk for major bleeding was significantly lower with DOACs compared with VKAs (RR 0.64, 95% CI 0.42–0.99, *I*^2^ = 0%). In cohort studies, DOAC was associated with a lower risk for all-cause death compared with VKAs; however, with high heterogeneity (RR 0.78, 95% CI 0.62–0.98, *I*^2^ = 80%). No significant differences were found regarding ischemic stroke or systemic embolism and gastrointestinal bleeding.

**Conclusion:**

In dialysis patients with nonvalvular AF, DOACs were associated with significantly reduced risk for major bleeding in RCTs and significantly reduced risk for all-cause death in cohort studies. These findings suggest that DOACs may provide a higher net clinical benefit compared with VKAs in dialysis patients.

**Graphical Abstract:**

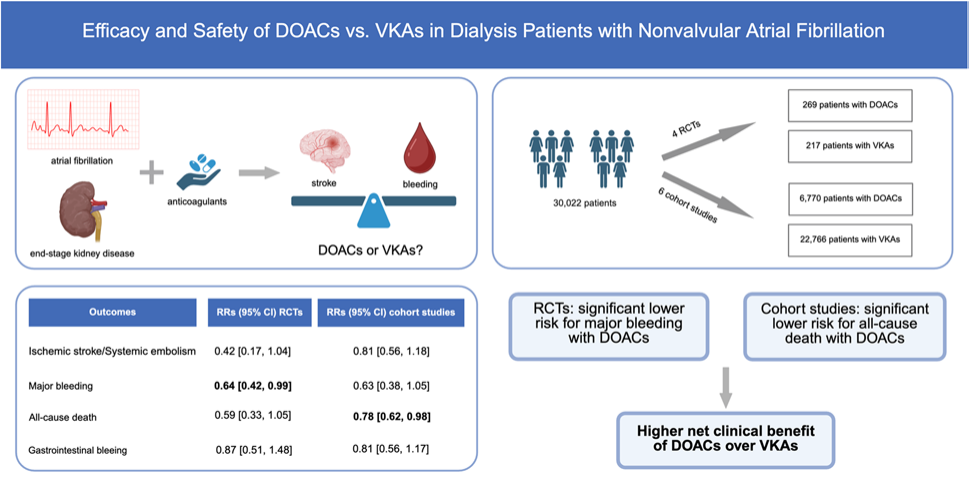

**Supplementary Information:**

The online version contains supplementary material available at 10.1007/s00392-025-02711-7.

## Introduction

Patients with chronic kidney disease (CKD), especially those with end-stage kidney disease (ESKD), are at increased risk for cardiovascular disease including cardiac arrhythmias such as atrial fibrillation (AF) [1, 2]. In patients with AF, the use of oral anticoagulants such as vitamin K antagonists (VKAs) reduces the risk for ischemic stroke or systemic embolism. Interaction between VKAs and pharmaceuticals or food compositions could elicit an instable hemostasis. Side effects such as calciphylaxis may cause ischemic cutaneous ulceration and lead to a 1-year mortality of 54% [3]. Direct oral anticoagulants (DOACs) directly inhibit the factor IIa or factor Xa, respectively, and improve outcomes in nonvalvular AF patients compared with VKAs [4].

Compared with patients with preserved renal function, dialysis patients are at a higher risk for both bleeding and thrombosis [5]. Dialysis patients were excluded in the landmark trials which investigated the efficacy and safety of DOACs in patients with nonvalvular AF [6–9]. VKAs are still the most commonly used anticoagulant in this population, but calciphylaxis predominantly affects dialysis patients [10], and the time in therapeutic range (TTR) is substantially reduced in dialysis patients despite comparable INR monitoring intensity [11].

Cohort studies focusing on the comparison of DOACs with VKAs in dialysis patients with nonvalvular AF reported inconsistent results [12, 13]. Randomized controlled trials (RCTs) were discontinued prematurely due to feasibility challenges primarily driven by slow recruitment, preventing them from achieving sufficient power [14, 15]. Systematic review and meta-analysis of existing data is needed to clarify the current state.

We conducted an updated systematic review and meta-analysis of both RCTs and cohort studies which focused on the efficacy and safety of DOACs and VKAs in dialysis patients with nonvalvular AF.

## Methods

This systematic review and meta-analysis followed the recommendation of Preferred Reporting Items for Systematic Reviews and Meta-Analyses (PRISMA) Guidelines [16] and was registered in the International Prospective Register of Systematic Reviews (PROSPERO) database (protocol number: CRD42024545428) on June 2, 2024.

### Data sources and search strategies

Literature research was done in PubMed, Embase, Cochrane, and ClinicalTrials.gov using MeSH and other complementary terms including chronic kidney failure, dialysis, direct oral anticoagulants, and vitamin K antagonists (supplement 1). All articles published in English were screened from inception to December 5, 2024.

Inclusion criteria:

- Study population: patients with ESKD on chronic dialysis and nonvalvular AF
- Comparison of DOACs with VKAs
- Outcomes included stroke or bleeding

Exclusion criteria:

- ESKD but not yet on dialysis
- Other indications than nonvalvular AF for anticoagulation

Ti. L. and To. L. reviewed the titles and abstracts independently for the first screening. In case of discrepancies, both authors read the full articles and decided the eligibility independently. In case of persistent discrepancies, C. O. was consulted for consensus. Both RCTs and cohort studies were included in this meta-analysis.

### Outcomes and data extraction

The outcomes of interest included the composite outcome of ischemic stroke or systemic embolism, major bleeding, all-cause death, and gastrointestinal bleeding.

The extracted study characteristics included the primary authors, study type, year of publication, data source, investigated drugs and dosages, sample sizes, time in therapeutic range (TTR) of VKAs, percentage of male sex, CHA2DS2-VASc score, HAS-BLED score, and follow-up period. If data for both intention-to-treat and per-protocol existed, the intention-to-treat analysis was used for this meta-analysis. The data extraction was performed by Ti. L. and To. L. independently.

### Study quality assessment

The risk of bias was assessed using the Risk of Bias-2 (RoB2) tool [17] for RCTs and the Risk of Bias in Non-randomized Studies—of Interventions, version 2 (ROBINS-I V2) tool [18] for cohort studies. The RoB2 tool contains five domains (risk of bias arising from the randomization process, risk of bias due to deviations from the intended interventions, missing outcome data, risk of bias in measurement of the outcome, and risk of bias in selection of the reported result) with three different judgements (low, high, or some concerns). The ROBINS-I V2 tool addressed seven domains: risk of bias due to confounding, risk of bias in classification of interventions, risk of bias in selection of participants into the study (or into the analysis), risk of bias due to missing data, risk of bias arising from measurement of the outcome, and risk of bias in selection of the reported result. In each domain, studies can be labeled with low, moderate, serious, or critical risk of bias.

### Statistical analysis

In this meta-analysis, we included both RCTs and cohort studies but analyzed the data separately. Quantitative analysis was conducted in RevMan (version 5.4.1; Nordic Cochrane Center [Cochrane Collaboration], Copenhagen, Denmark). We synthesized the dichotomous outcomes to obtain the risk ratios (RRs) with 95% confidence intervals (CIs). The forest plots were produced in the software using the random-effect model and the Mantel–Haenszel method. *I*^2^ statistics were used to assess statistical heterogeneity. *I*^2^ < 25% was considered low, *I*^2^ between 26 and 50% as moderate, *I*^2^ between 51 and 75% as substantial, and *I*^2^ > 75% as high heterogeneity. Heterogeneity was regarded as statistically significant if the *P* value was lower than 0.05. Funnel plots were constructed for publication bias.

## Results

### Literature search

**Fig. 1:**
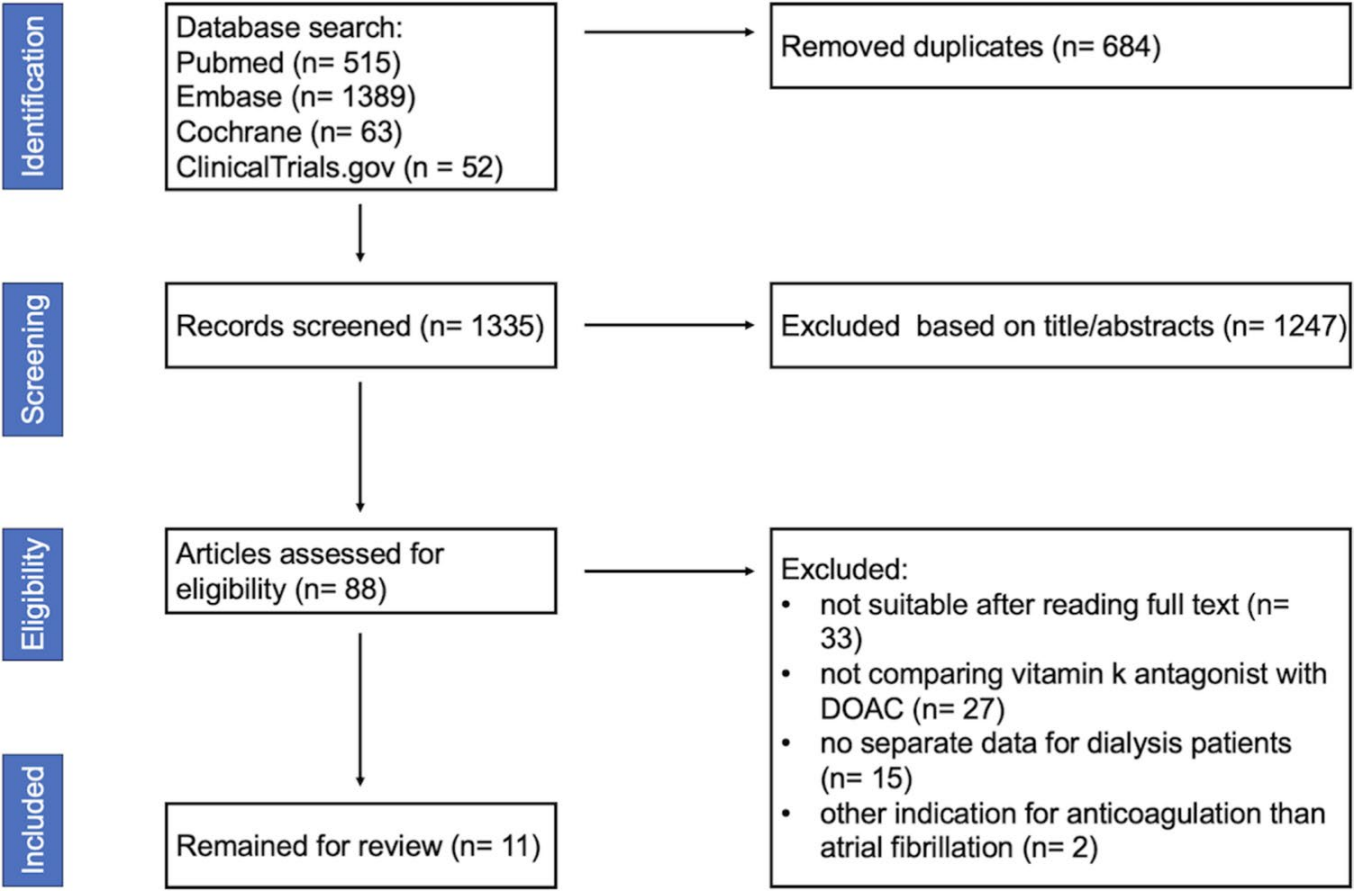
Literature search was done in PubMed, Embase, Cochrane Library, and ClinicalTrial.gov. Eleven studies remained for systematic review

In the initial literature search, we identified a total of 2019 publications with 515, 1,389, 63, and 52 publications in PubMed, Embase, Cochrane Library, and ClinicalTrials.gov, respectively. After removing 684 duplicates, we screened 1335 publications and excluded 1247 based on titles or abstracts. After reading full texts, 77 publications were excluded for not meeting inclusion or meeting exclusion criteria: 33 were not focusing on anticoagulation in dialysis patients, 27 did not compare VKAs with DOACs, 15 did not offer separate data for dialysis patients, and 2 included patients with other indications for anticoagulation than AF. Ultimately, 11 publications underwent systematic review. The detailed flow diagram is shown in Fig. 1.

### Studies and study population

Of the 11 eligible publications, 4 were RCTs [14, 15, 19, 20] and 7 were cohort studies [12, 13, 21–25]. Six studies investigated apixaban [13–15, 20, 21, 24], 2 studies investigated rivaroxaban [12, 19], 1 study had dabigatran as a subgroup [12], and 3 studies pooled the data of all DOACs together [22, 23, 25]. Of the two studies investigating rivaroxaban, De Vriese et al. used a dosage of 10 mg once daily [19], whereas the patients in the study by Chan et al. received either 15 mg or 20 mg once daily [12]. Among the three RCTs evaluating apixaban, Pokorney et al. applied the standard labeled dosage (5 mg twice daily or 2.5 mg twice daily for patients meeting at least two of the following criteria: creatinine ≥ 1.5 mg/dL, age ≥ 80 years, or body weight ≤ 60 kg) [15]. Harel et al. also applied the standard dosage but permitted the clinicians to reduce it at their discretion if deemed inappropriate [20]. Reinecke et al. administrated a reduced dose of 2.5 mg twice daily for all patients [14]. Among the three cohort studies, Wetmore et al. reported separate data for patients treated with apixaban 5 mg twice daily and those receiving apixaban only 2.5 mg twice daily, although the full dose was indicated [13]. Siontis et al. and Moore et al. conducted subgroup analysis comparing patients treated with 5 mg and 2.5 mg twice daily, without taking into consideration whether the dosages used were label-concordant [21, 24]. The detailed DOAC types and dosages of each study are listed in supplement 8. Within the studies, the study population was well balanced between the DOACs and VKAs groups regarding the ages, percentage of males, CHA2DS2-VASc score, and HAS-BLED score. The detailed characteristics of the studies are listed in Table 1.

**Table 1:** Characteristics of included studies

Studies	Year of publication	Data source	Sample size (*n*)	TTR of VKAs (%)***	Age (year)***	Male (%)	CHA2DS2-VASc score***	HAS-BLED score***	Follow-up period***
**RCTs**									
De Vriese et al	2021	02/2015–07/2019 Belgien	Rivaroxaban 10 mg (46)	59 [55–63]	79.9 [74.4–83.9]	35 (76.1)	4.7 (1.4), 5 [4–5]	4.6 (0.8), 4 [4–5]	1,88 [1.01–3.38] years
			Rivaroxaban 10 mg + VK2** (42)		79.6 [73.2–83.1]	28 (66.7)	4.5 (1.4), 4 [4–5]	4.7 (0.9), 5 [4–5]	
			Warfarin (44)		80.3 [71.5–84.3]	25 (56.8)	4.8 (1.5), 5 [4–6]	4.7 (0.9), 5[4–5]	
Pokorney et al	2022	01/2017–01/2019 USA	Apixaban LC** (82)	44 [23–59]	69.0 [61.0–76.0]	48 (48.5)	4 [3–5]	na	330 (median)
			Warfarin (72)		68.0 [60.5–72.5]	50 (69.4)	4 [3–5]		340 (median)
Reinecke et al	2023	06/2017–05/2022 Germany	Apixaban 2 × 2.5 mg (48)	50.7 (median)	76.5 [68–81]	31 (64.6)	4.5 (1.62), 5.0 [3.5–5.0]	4.25 (1.02), 4.0 [3.5–5.0]	429 [174–702]
			Phenprocoumon (49)		77 [70–80]	37 (75.5)	4.54 (1.49), 4.5 [4–6]	4.15 (1.03), 4.0 [3.5–5.0]	506 [289–702]
Harel et al	2024	12/2019–06/2022 Canada & Australia	Apixaban MD** (51)	58.1 [36.6–69.7]	72 [64–81]	37 (72)	4 [3–5]	na	6 months
			Warfarin (52)		71 [67–78]	43 (83)	4 [3–5]		6 months
**Cohort studies**									
Chan et al	2015	10/2010–10/2014 USA FMCNA* ESRD database	Rivaroxaban MD (244)	13.7% of patients TTR > 60%	66.9 (12)	148 (60.5)	2.2 (1.0)	na	72 py**
			Dabigatran MD (281)		68.4 (12)	166 (59.2)	2.3 (1.0)		123 py
			Warfarin (8,064)		70.6 (11)	4,935 (61.2)	2.4 (1.0)		3839 py
Siontis et al	2018	10/2010–12/2015 USA USRDS*	Apixaban HD** (1,034)	na	65.16 (10.14)	616 (59.6)	4.92 (1.71)	na	Until 12/2015 or death or censoring
			Apixaban LD** (2,351)		71.79 (11.65)	1,664 (50.4)	5.54 (1.77)		
			Warfarin (7,053)		68.14 (11.80)	3,796 (53.8)	5.27 (1.77)		
See et al	2020	06/2012–12/2017 Taiwan NHIRD*	DOACs (448)	na	74.3 (10.9)	222 (49.6)	4.5 (1.9)	3.7 (1.1)	From the index date until the first occurrence of any study outcome or the end date of the study period
			Warfarin (448)		75.2 (10.9)	213 (47.5)	4.7 (1.9)	3.6 (1.2)	
Wetmore et al	2022	04/2013–12/2018 USA USRDS	Apixaban HD (2,382)	na	66.2 (9.4)	1,462 (61.4)	4.3 (1.7)	2.9 (0.8)	Until the earliest of the following: date of the outcome of interest, death, loss of Medicare coverage, change of dialysis modality, receipt of a kidney transplant, or other loss of follow-up for the intention-to-treat (ITT) analysis
			Apixaban UD** (2,257)			1,293 (57.3)	4.7 (1.7)	3.1 (0.8)	
			Warfarin (12,517)			7,823 (62.5)	4.5 (1.7)	3.0 (0.8)	
Moore et al	2024	01/2018–12/2021 USA Academic institution	Apixaban MD (53)	41.65 [29.75–55.52]	68.74 (10.28)	29 (54.7)	3 [3–4]	2 [2–3]	2 years
			Warfarin (57)		63.37 (16.18)	30 (54.5)	3 [2–4]	3 [0–4]	
Laville et al	2024	01/2012–12/2020 French REIN* and SNDS*	DOACs (184)	na	74 [67–81]	(64)	na	na	Until the earliest of following events: date of the outcome of interest, death, kidney transplantation, discontinuation of dialysis or loss to follow-up 1,7 [0.8–3.2] years
			VKAs (2,317)		73 [64–81]	(63)	na	na	
Roh et al	2024	09/2012–08/2020 South Korea HIRA*	DOACs (106)	na	67.8 (9.4)	(67.9)	2.6 (1.4)	3.3 (1.1)	3.6 [2.1–5.5] years
			Warfarin (319)		66.2 (10.7)	(65.9)	2.5 (1.5)	3.1 (1.2)	

^*^FMCNA, Fresenius Medical Care of North America; *USRDS*, United States Renal Data System; *NHIRD*, National Health Insurance Research Database; *REIN*, Renal Epidemiology and Information Network; *SNDS*, Système National des Données de Santé; *HIRA*, Health Insurance Review and Assessment Service

^**^VK2, vitamin K2; py, patient year; na, not available

*LC* label-concordant dose (5 mg twice daily, or 2.5 mg twice daily if meeting at least 2 of the following criteria: creatinine ≥ 1.5 mg/dL, age ≥ 80 years, or body weight ≤ 60 kg)

*HD* high dose (5 mg twice daily)

*LD* low dose (2.5 mg twice daily)

*MD* mixed dosage

*UD* underdosage (2.5 mg twice daily, although 5 mg twice daily indicated)

^***^If not otherwise stated, the data are shown as mean (SD) or median [Q1–Q3]

From the 11 eligible studies, 10 were included in the meta-analysis. See et al. [23] was excluded because they did not present the results with absolute event numbers. Pokorney et al. [15] and Reinecke et al. [14] were terminated early because of slow recruitment, so the data should be interpreted with caution. De Vriese et al. [19] had three arms receiving warfarin, rivaroxaban, and rivaroxaban with vitamin K2 supplement. In this meta-analysis, we pooled the data of the latter two arms to the DOACs group. De Vriese et al. [19] distinguished life-threatening bleeding from major bleeding. For this analysis, the event numbers were pooled to major bleeding. Siontis et al. [21] and Wetmore et al. [13] used the data from United States Renal Data System (USRDS) with overlapping periods, so that we only used the data from 2016 to 2018 from Wetmore et al. Laville et al. [25] included dialysis patients with various indications for anticoagulation therapy, and we only used the data from patients with a history of nonvalvular AF. Regarding the limited data and inconsistent DOAC dosing across the included studies, it was not feasible to perform a network meta-analysis for each individual DOAC or to analyze different dosages of DOACs separately. Therefore, we focused on comparing of the underlying mechanisms of the two drug classes and pooled the data of various DOAC types and dosages together. Due to different forms with which results were provided, the numbers of patients who experienced the events were used in RCTs and the numbers of occurred events were used in cohort studies. The study outcomes are listed in Table 2.

**Table 2:** Study outcomes

**Study**	**Group (*n*)**	All-cause death*	Ischemic stroke*	Systemic embolism*		Major bleeding*	Gastro-intestinal bleeding*	**Included for meta-analysis**
**RCTs**								
De Vriese et al	Rivaroxaban 10 mg (46)	30	4	0		8	9	Yes
	Rivaroxaban 10 mg + VK2** (42)	27	2	0		9	13	
	Warfarin (44)	32	7	0		17	12	
Pok-orney et al	Apixaban LC** (82)	21	1	na**		9	4	Yes
	Warfarin (72)	43	2	na		7	6	
Reinecke et al	Apixaban 2 × 2.5 mg (48)	9	0	na		5	na	Yes
	Phenprocoumon (49)	12	1	na		6	na	
Harel et al	Apixaban MD** (51)	2	0	na		2	1	Yes
	Warfarin (52)	9	0	na		4	0	
**Cohort studies**								
Chan et al	Rivaroxaban MD (244)	na	8	0		46	19	Yes
	Dabigatran MD (281)	na	11	2		106	48	
	Warfarin (8,064)	na	221	25		1,858	1,016	
Siontis et al	Apixaban HD (1,034)	48	26			54	70	Yes
	Apixaban LD (1,317)	111	55			75	85	
	Warfarin (7,053)	753	373			715	710	
See et al	DOACs (448)	na	6.67/100 py**			7.07/100 py	5.51/100 py	No
	Warfarin (448)	na	5.30/100 py			7.15/100 py	5.20/100 py	
Wetmore et al	Apixaban HD (2,382)	716	52			127	na	Yes
	Apixaban UD** (2,257)	741	54			117		
	Warfarin (12,517)	6,090	424			1,226		
Wetmore et al 2016–2018	Apixaban HD (1,774)	407	31			72	na	Yes
	Apixaban UD (1,777)	470	34			76		
	Warfarin (4,956)	1,559	104			334		
Moore et al	Apixaban MD (53)	27	4		na	9	na	Yes
	Warfarin (57)	25	6		na	11		
Laville et al	DOACs (184)	na	na		na	8	na	Yes
	VKAs (2,317)					1,437		
Roh et al	DOACs (106)	na	1		na	na	5	Yes
	Warfarin (319)	na	25		na	na	18	

^*^If not otherwise stated, the numbers refer to patient number in RCTs and event number in cohort studies

^**^VK2, vitamin K2; py, patient year; na, not available

*LC* label-concordant dose (5 mg twice daily, or 2.5 mg twice daily if meeting at least 2 of the following criteria: creatinine ≥ 1.5 mg/dL, age ≥ 80 years, or body weight ≤ 60 kg)

*HD* high dose (5 mg twice daily)

*LD* low dose (2.5 mg twice daily)

*MD* mixed dosage

*UD* underdosage (2.5 mg twice daily, although 5 mg twice daily indicated)

### Bias assessment

An overview of all the bias assessments is listed as supplement 2 and 3. All four RCTs [14, 15, 19, 20] were labeled with a low risk of bias. From the cohort studies, five [13, 21–23, 25] of them were also labeled with a low risk of bias. However, Chan et al. [12] and Moore et al. [24] were evaluated as having a moderate risk of bias in the domain “selection of the reported result,” thus resulting in a moderate overall risk of bias. The funnel plots are listed as supplement 4. However, the number of included studies was too low to detect asymmetry with sufficient power.

### Outcomes

This meta-analysis included 7,039 patients under DOACs and 22,983 patients under VKAs, of which 269 patients under DOACs and 217 patients under VKAs were recruited in RCTs.

### Ischemic stroke or systemic embolism

**Fig. 2:**
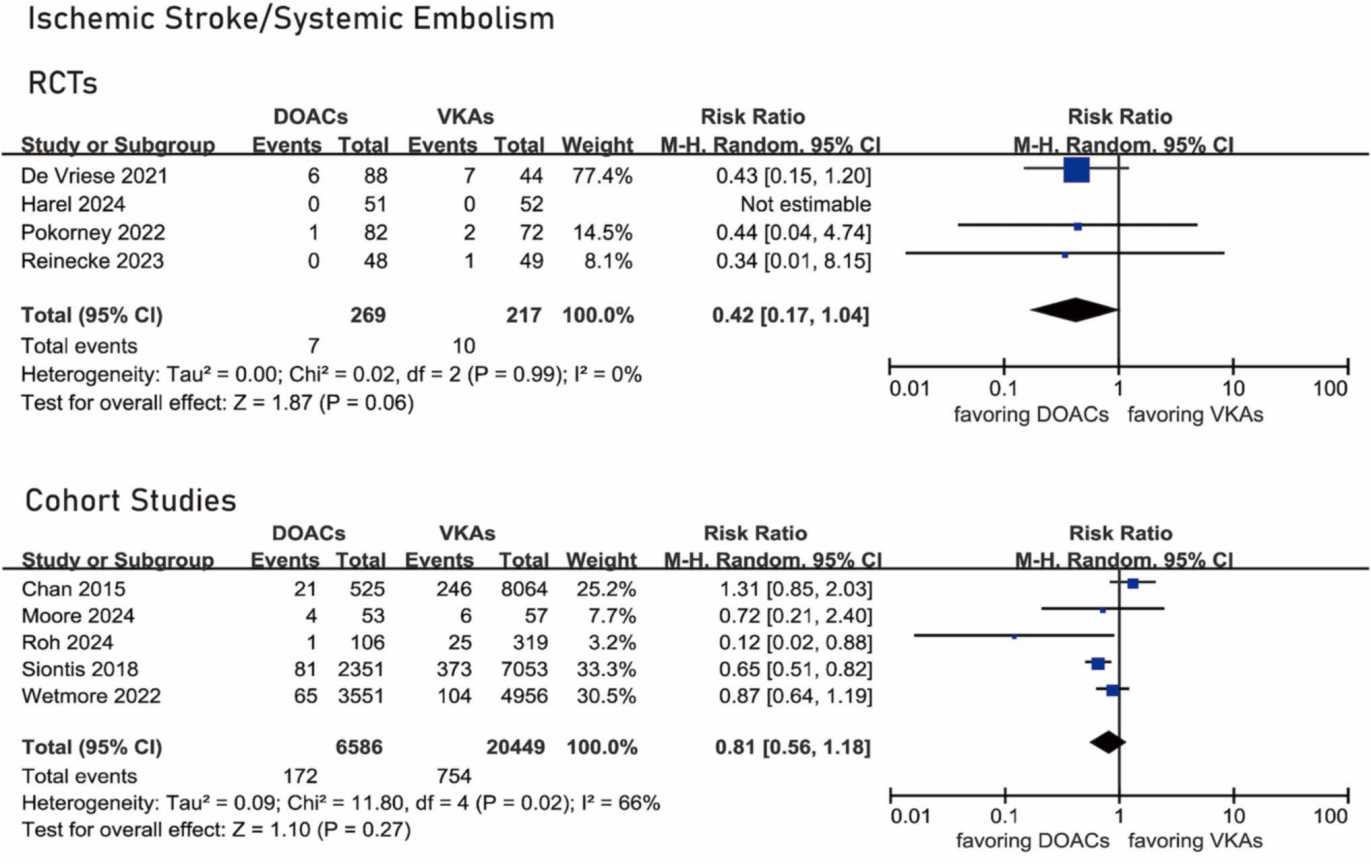
Forest plots of the composite outcome of ischemic stroke or systemic embolism

All RCTs [14, 15, 19, 20] provided data about the composite outcome of ischemic stroke or systemic embolism. Ischemic stroke or systemic embolism occurred in 7 of 269 patients under DOACs and 10 of 217 patients under VKAs. There was a trend for lower risk for the composite outcome under DOACs compared with VKAs, however, without statistical significance (RR 0.42, 95% CI 0.17–1.04, *I*^2^ = 0%, *P* 0.99) (Fig. 2).

Five cohort studies [12, 13, 21, 22, 24] provided data about the composite outcome of ischemic stroke or systemic embolism, which occurred in 172 of 6586 patients under DOACs and in 754 of 20,449 patients under VKAs. There was no statistically significant difference between the two groups (RR 0.81, 95% CI 0.56–1.18, *I*^2^ = 66%, *P* 0.02) (Fig. 2).

### Major bleeding

**Fig. 3:**
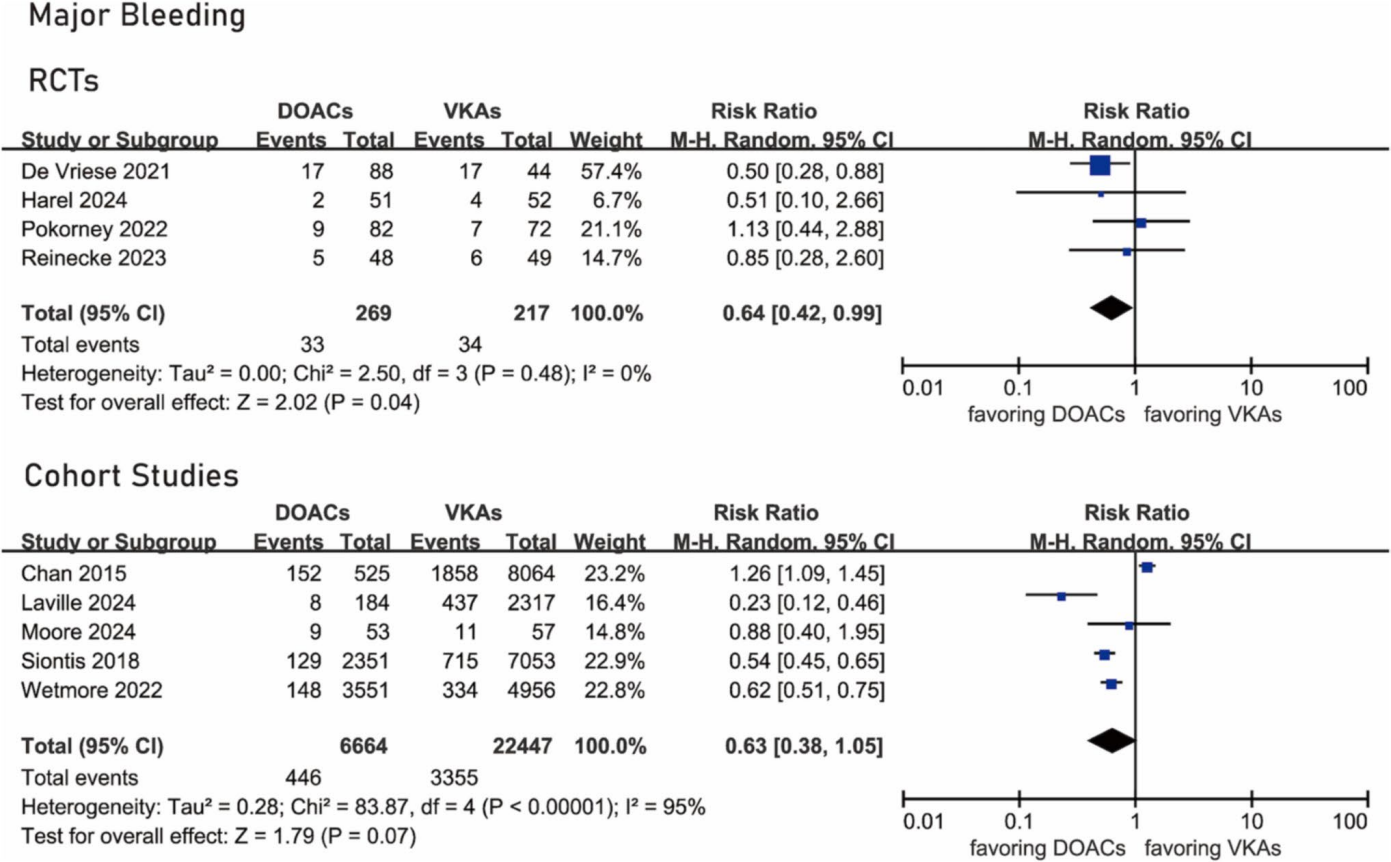
Forest plots of major bleeding

All RCTs [14, 15, 19, 20] provided data about major bleeding. Major bleeding occurred in 33 of 269 patients under DOACs and 34 of 217 patients under VKAs. The rate of major bleeding was significantly lower in patients under DOACs compared with VKAs (RR 0.64, 95% CI 0.42–0.99, *I*^2^ = 0%, *P* 0.48) (Fig. 3).

Five cohort studies [12, 13, 21, 24, 25] provided data about major bleeding. Major bleeding occurred in 446 of 6,664 patients under DOACs and in 3,355 of 22,447 patients under VKAs. No significant difference existed between the two groups (RR 0.63, 95% CI 0.35–1.05, *I*^2^ = 95%, *P* < 0.00001) (Fig. 3).

### All-cause death

**Fig. 4:**
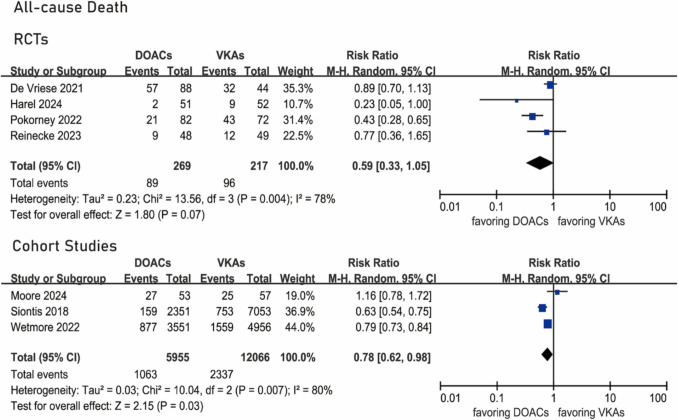
Forest plots of all-cause death

All RCTs [14, 15, 19, 20] provided data about all-cause death. All-cause death occurred in 89 of 269 patients under DOACs and 96 of 217 patients under VKAs. There was no significant difference in the rate of all-cause death (RR 0.59, 95% CI 0.33–1.05, *I*^2^ = 78%, *P* 0.004) (Fig. 4).

Three cohort studies [13, 21, 24] provided data about all-cause death. All-cause death occurred in 1,063 of 5,955 patients under DOACs and in 2,337 of 12,066 patients under VKAs. The heterogeneity in the distribution of the data was high. The use of DOACs was significantly associated with a decreased risk for all-cause death compared with VKAs (RR 0.78, 95% CI 0.62–0.98, *I*^2^ = 80%, *P* 0.007) (Fig. 4).

### Gastrointestinal bleeding

**Fig. 5:**
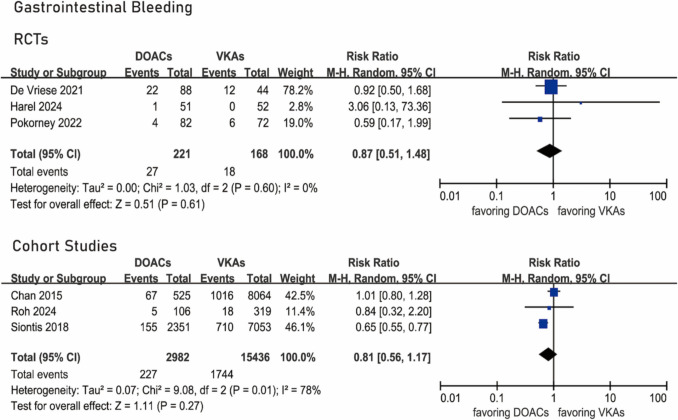
Forest plots of gastrointestinal bleeding

Three RCTs [15, 19, 20] provided data about gastrointestinal bleeding. Gastrointestinal bleeding occurred in 27 of 221 patients under DOACs and 18 of 168 patients under VKAs. No significant difference was identified (RR 0.87, 95% CI 0.51–1.48, *I*^2^ = 0%, *P* 0.60) (Fig. 5).

Three cohort studies [12, 21, 22] provided data about gastrointestinal bleeding. Gastrointestinal bleeding occurred in 227 of 2,982 patients under DOACs and in 1,744 of 15,436 patients under VKAs. No significant difference existed (RR 0.81, 95% CI 0.56–1.17, *I*^2^ = 78%, *P* 0.01) (Fig. 5).

### Subgroup analysis

Taking the bias assessment into consideration, we conducted an analysis including only publications with low overall risk of bias and excluded Chan et al. [12] and Moore et al. [24]. The risks for major bleeding (RR 0.51, 95% CI 0.37–0.69, *I*^2^ = 75%, *P* 0.02), all-cause death (RR 0.71, 95% CI 0.58–0.88, *I*^2^ = 82%, *P* 0.02), and gastrointestinal bleeding (RR 0.66, 95% CI 0.56–0.78, *I*^2^ = 0%, *P* 0.63) were significantly lower in patients under DOACs in the cohort studies, while no significant difference existed regarding the composite outcome of ischemic stroke or systemic embolism (RR 0.70, 95% CI 0.47–1.03, *I*^2^ = 63%, *P* 0.07). The forest plots are shown in supplement 5a-d.

In another subgroup analysis, we only included studies focusing on apixaban, since this was the predominant DOAC tested across these trials. In three RCTs [14, 15, 20], the risk for all-cause death (RR 0.48, 95% CI 0.30–0.77, *I*^2^ = 25%, *P* 0.26) was significantly lower in patients receiving apixaban, while the differences in the risks for the composite outcome of ischemic stroke or systemic embolism (RR 0.40, 95% CI 0.06–2.69, *I*^2^ = 0%, *P* 0.90) and major bleeding (RR 0.90, 95% CI 0.47–1.74, *I*^2^ = 0%, *P* 0.71) were not statistically significant. In three cohort studies [13, 21, 24], the risks for the composite outcome of ischemic stroke or systemic embolism (RR 0.73, 95% CI 0.60–0.89, *I*^2^ = 9%, *P* 0.71), major bleeding (RR 0.58, 95% CI 0.51–0.67, *I*^2^ = 2%, *P* 0.36), and all-cause death (RR 0.78, 95% CI 0.62–0.98, *I*^2^ = 80%, *P* 0.007) were all significantly lower in patients under DOACs compared with VKAs. The forest plots are shown in supplement 6a-c.

## Discussion

This meta-analysis provides insight into the efficacy and safety of DOACs and VKAs in dialysis patients with nonvalvular AF. The main findings of this meta-analysis were as follows: (1) In RCTs, the risk for major bleeding in patients under DOACs was significantly lower compared to VKAs, whereas the risk for ischemic stroke or systemic embolism, all-cause death, and gastrointestinal bleeding was similar between the two groups. (2) In cohort studies, dialysis patients under DOACs had a significantly lower rate of all-cause death, while there were no significant differences regarding ischemic stroke or systemic embolism, major bleeding, and gastrointestinal bleeding. However, the results of cohort studies should be interpreted with caution due to high heterogeneity.

Since pooling the data from RCTs and cohort studies could jeopardize the validity of results due to noticeable lower weight of RCTs despite significantly higher data quality, we analyzed four RCT studies and six cohort studies separately. The heterogeneity enhanced the importance of separate data analysis. The RCTs showed very low heterogeneity. In cohort studies, the heterogeneity was high across all four outcomes.

The increased risks for both bleeding and thromboembolism and the high susceptibility to adverse events of dialysis patients lead to a dilemma in anticoagulation therapy and add challenges to studies focusing on this topic. Existing data suggest that DOACs might have a more favorable risk–benefit profile compared with VKAs. The bleeding risk of dialysis patients is substantially increased due to platelet dysfunction and altered platelet-vessel wall interactions [26]. Accumulation of anticoagulants due to reduced renal elimination could further worsen this problem. In the meta-analysis of RCTs, the risk for major bleeding was significantly reduced in patients under DOACs compared with VKAs, demonstrating the safety of DOACs in dialysis patients. Activation of the coagulation cascade in dialysis patients increases the risk for thromboembolism [27]. In this meta-analysis of both RCTs and cohort studies, the use of DOACs was numerically associated with a reduced risk for ischemic stroke or systemic embolism; however, without statistical significance. The lack of significance may stem from studies being halted prematurely [14, 15] and being underpowered [28]. DOACs also showed a trend for a lower risk for all-cause death compared with VKAs. However, since dialysis patients are mostly elderly and multimorbid, there might be other reasons than bleeding or stroke causing the high mortality, independent of anticoagulation. These findings were in concordance with results discussed by See et al. [23], suggesting no significant differences regarding ischemic stroke or systemic embolism, major bleeding, and gastrointestinal bleeding.

Of the 11 included studies, 5 studies [14, 15, 20, 23, 24] provided data about the median TTR of patients under VKAs with values of 42–59%, which were mostly better than the overall TTR of 42.4% of dialysis patients [11], enhancing the possible net benefit of DOACs over VKAs in the real world. In Chan et al. [12], only 13.7% of the patients under VKAs reached a TTR of at least 60%, which was lower than the overall rate of 21.1% of dialysis patients [11]. In the subgroup analysis of only patients with TTR ≥ 60% in Chan et al. [12], dabigatran and rivaroxaban were still associated with an increased risk for major bleeding compared with warfarin, which was consistent with the primary results including all warfarin patients. Regarding the aspects above, there is no obvious evidence showing the influence of therapy management of VKAs on the results of this meta-analysis.

In the subgroup analysis of all studies with low overall risk of bias, treatment with DOACs was associated with significantly lower risks for major bleeding, all-cause death, and gastrointestinal bleeding, while in the primary analysis, only all-cause death demonstrated a significance favoring DOACs. The higher-quality data that tend to favor DOACs also provide indirect support for their net clinical benefit over VKAs.

Due to the lack of strong evidence regarding the safety and efficacy of DOACs in dialysis patients, DOACs are contraindicated for stroke prevention in dialysis patients with atrial fibrillation in the European Union (EU)[29]. Nonetheless, the use of DOAC as off-label therapy has increased over the years [25]. Of all the DOACs approved for stroke prevention in patients with nonvalvular AF and preserved renal function, apixaban shows the lowest renal elimination [30]. Based on pharmacokinetic data, the Food and Drug Administration (FDA) approved the use of apixaban in dialysis patients at a standard dosage of 5 mg twice daily, with dose adjustment if the patient meets one of the following criteria: body weight ≤ 60 kg or age ≥ 80 years [31]. In the subgroup analysis focusing only on apixaban, apixaban also showed a more favorable risk–benefit profile, associated with significantly lower risk for all-cause death in RCTs and significantly lower risks across all the investigated outcomes in cohort studies. This is also in concordance with Zhang et al. [32], who showed significantly reduced risk for major bleeding and clinically relevant non-major bleeding under apixaban in cohort studies. The heterogeneity in the subgroup analysis was much lower compared with the primary analysis, indicating the reliable benefit of apixaban over VKAs. The reduction of I^2^ indicated that the high heterogeneity in the primary analysis might be partially caused by the different types of DOACs. Given the favorable outcomes and the comparatively larger body of available data, apixaban appears to be the most appropriate choice among DOACs for patients on dialysis.

Although the FDA approved a standard dose of apixaban for dialysis patients, the dosages administrated in the included studies were inconsistent. All three cohort studies performed subgroup analyses based on different apixaban doses [13, 21, 24]. In the study by Wetmore et al., no significant differences were observed between patients treated with standard and reduced doses regarding stroke, systemic embolism, or major bleeding. However, patients on the standard dose showed a significantly lower risk for all-cause mortality compared with those on warfarin, while the mortality difference between reduced dose apixaban and warfarin was not significant [13]. In the study by Siontis et al., both standard and reduced doses were associated with lower major bleeding risks compared with warfarin, but only the standard dose was associated with reduced thromboembolic events and mortality [21]. Moore et al. found that the 5 mg twice-daily dose was not associated with an increased risk for bleeding, whereas stroke events occurred only in patients receiving apixaban 2,5 mg twice daily [24]. Taken together, the findings indicate that the standard dose of apixaban may prevent more thromboembolic events without increasing the risk of bleeding, making it potentially more suitable for dialysis patients.

Across all included studies, the incidence of major bleeding exceeded that of stroke or systemic embolism, regardless of the type of anticoagulation therapy used, which raises concerns about the net benefit of anticoagulation therapy in this population. Harel et al. conducted a pilot RCT comparing the safety and efficacy of apixaban, warfarin, and no anticoagulation, with 51, 52, and 48 patients in each group, respectively [20]. Among all the patients, only 1 patient in the no-anticoagulation group experienced stroke. Major bleeding was reported in 2, 4, and 2 patients in the apixaban, warfarin, and no-anticoagulation groups, respectively, and death occurred in 2, 9, and 4 patients, respectively, suggesting that no anticoagulation was not inferior to warfarin. However, due to the small sample size, the study was underpowered to detect statistically significant differences. In a network meta-analysis by Kao et al., DOACs, VKAs, and no anticoagulation were compared [33]. VKAs were associated with a significantly higher risk of major bleeding, while no differences were found for thromboembolism or all-cause death, suggesting that no anticoagulation might be a noninferior alternative in patients with a high bleeding risk. Further RCTs directly comparing the three different regimes are needed to achieve a robust conclusion.

Another alternative for patients with contraindications to long-term anticoagulation or a high bleeding risk is left atrial appendage occlusion (LAAO). A meta-analysis comparing the efficacy and safety of LAAO with DOACs in patients with AF showed a superior profile of LAAO over DOACs in terms of all-cause death, cardiovascular events, and major bleeding [34]. For patients with ESKD, LAAO was also associated with reduced mortality compared with warfarin or no anticoagulation in a multi-center, prospective observational study [35]. However, studies directly comparing DOAC with LAAO in ESKD patients with AF are still lacking, which represents an important perspective for future studies. The ongoing randomized controlled LAA-KIDNEY trial (NCT05204212), which compares LAAO with conservative therapy in patients with end-stage kidney disease (ESKD), may provide more robust evidence in the future [36]. In addition to factor Xa or factor IIa inhibitors, factor XIa inhibitor has also been developed, which should prevent thrombotic events without significantly increasing the risk of bleeding [37]. A phase 2 trial showed lower bleeding rates of factor XIa inhibitor compared with standard dose apixaban in patients with AF [38]. Several phase 3 trials are currently ongoing and would offer new opportunities for dialysis patients.

### Strengths and limitations

Several other meta-analyses have been published on this topic, but none has settled the matter. Kyriakoulis et al. [39] performed a meta-analysis comparing DOACs with VKAs in patients with AF and ESKD on hemodialysis, in which data from three RCTs and three cohort studies were pooled and analyzed together despite the lower weight of RCTs. Furthermore, one of the included cohort studies, Lin et al. [40], investigated also ESKD patients not on dialysis, which might have impacted the final results. Another cohort study from Wetmore et al. [13], however, was not included. Kao et al. [33] used repeated data from Wetmore et al. [13] and Siontis et al. [21], both of which extracted data from USRDS with overlapping periods between April 2013 and December 2015. Mapili et al. [41] conducted a meta-analysis of 3 RCTs and did not include the data of patients receiving rivaroxaban and vitamin K2 supplement from De Vriese et al. [19]. Since vitamin K2 should not influence the hemostasis, it is questionable whether the exclusion of this group was reasonable, especially when the available data were already very small. In our meta-analysis, we included new studies and took the concerns above into consideration.

There are several limitations of this meta-analysis. Firstly, due to limited available data, we focused primarily on the different mechanisms of action between DOACs and VKAs and pooled the data of different DOAC types and dosages together. Given that renal elimination and pharmacokinetic profiles vary among individual DOACs, this may have influenced the results. This was also confirmed by the lower heterogeneity and more favorable outcomes observed in the subgroup analysis of apixaban.

Secondly, we pooled all the events of major bleeding together, although the authors defined major bleeding variously. Of the four RCTs, Reinecke et al. [14], Pokorney et al. [15], and Harel et al. [20] defined major bleeding according to the International Society on Thrombosis and Haemostasis (ISTH) [42]. In the study by De Vriese et al. [19], the pooled outcomes of major bleeding and life-threatening bleeding deviated only slightly from the definition of ISTH, so that the variable definition of major bleeding is unlikely to have a significant impact on the analysis of RCTs. Of the six cohort studies, Moore et al. [24] defined major bleeding according to ISTH, Wetmore et al. [13] used a similar definition, while Chan et al. [12] and Laville et al. [25] primarily focused on bleeding resulting in hospitalization. Siontis et al. [21] defined major bleeding as bleeding at a critical site, identified using ICD or procedure codes, which have limited positive predictive value and sensitivity depending on bleeding type and code position. [43] Roh et al. [22] reported intracranial hemorrhage and gastrointestinal bleeding as separate outcomes, without providing a composite definition of major bleeding. The inconsistent definitions of major bleeding might have influenced the results and also partly explained the high heterogeneity in cohort studies. An overview of the detailed definitions of major bleeding is listed as supplement 7.

Thirdly, there was a certain degree of methodological heterogeneity among the included studies. Outcomes were reported differently across study types. In RCTs, each outcome was counted once per patient, whereas in cohort studies, the same outcome could be counted multiple times. This repeated counting could have influenced the results, which is also one of the reasons why we analyzed RCTs and cohort studies separately. Although we performed separate analyses for the cohort studies, substantial methodological heterogeneity remained among them. While the follow-up periods of DOACs and VKAs population were generally well balanced, the different follow-up periods across the studies may have influenced the final results—particularly when the same outcomes were counted several times in the same patients. Since the retrospective data analysis in cohort studies was based on existing data, the study populations—as well as the completeness and quality of the documented information—may vary significantly across countries and databases. In addition to the pooling of different types and dosages of DOACs and inconsistent outcome definitions discussed above, methodological heterogeneity likely contributed to the high heterogeneity observed in the meta-analysis of cohort studies. Therefore, the results of cohort studies should be interpreted with caution. For future research, it would be advisable to use standardized outcome definitions (e.g., bleeding as defined by ISTH [42] or stroke as defined by Hicke et al. [44]) and to harmonize follow-up durations in order to enhance comparability and facilitate the synthesis of findings across studies.

### Current ongoing studies

A literature search identified three ongoing studies on anticoagulation in dialysis patients with nonvalvular AF. The APIDP2 study (NCT06045858) compares the safety and efficacy of apixaban and warfarin in nonvalvular AF patients on peritoneal dialysis [45]. The SACK study (NCT05679024) evaluates apixaban versus no anticoagulation in ESKD patients including those on dialysis [46]. The VISIONAIRE study (NCT06402851) compares edoxaban, warfarin, and no anticoagulation in ESKD patients with AF [47]. These studies will provide valuable data on anticoagulation strategies in dialysis patients with nonvalvular AF.

## Conclusion

This meta-analysis analyzed data from four RCTs and six cohort studies comparing DOACs with VKAs in dialysis patients with nonvalvular AF. In RCTs, DOACs significantly reduced the risk for major bleeding compared with VKAs. The risks for the composite outcome of ischemic stroke or systemic embolism, all-cause death, and gastrointestinal bleeding were similar between both groups. In cohort studies, DOAC use was significantly associated with a lower risk for all-cause death compared with VKAs. The risks for the composite outcome of ischemic stroke or systemic embolism, major bleeding, and gastrointestinal bleeding were not significantly different. Taken together, DOACs may have a higher net clinical benefit over VKAs. The high heterogeneity of the cohort studies was an important limitation, so that the findings should be interpreted with caution.

## Supplementary Information

Below is the link to the electronic supplementary material.

Supplementary file1 (PDF 2125 KB)

## Data Availability

The data underlying this article are available in the article and in its online supplementary material.
