# Supplementary material for: Comparing the efficacy and safety of direct oral anticoagulants with vitamin K antagonists in dialysis patients with nonvalvular atrial fibrillation: a systematic review and meta-analysis
Source: Clin Res Cardiol. 2025 Jul 28;115(6):965–79. doi: 10.1007/s00392-025-02711-7 (PMC13160957; doi:10.1007/s00392-025-02711-7)
Supplement: Supplementary file 1 — Supplementary file1 (PDF 2125 KB) [file 392_2025_2711_MOESM1_ESM.pdf]

SUPPLEMENTARY DATA

Article title: Comparing the Efficacy and Safety of Direct Oral Anticoagulants with Vitamin K Antagonists in Dialysis Patients with Nonvalvular Atrial Fibrillation: a Systematic Review and Meta-analysis

Journal: Clinical Research in Cardiology

Authors: Tian Li[1], Tong Li[2], Yujun Xu[3], Diona Gjermeni[1], Lukas Heger[1], Dirk Westerman[1], Christoph B. Olivier[1]

[1] Department of Cardiology and Angiology, University Heart Center Freiburg – Bad Krozingen, Faculty of Medicine, University of Freiburg, Freiburg, Germany;

[2] Department of Cardiac Surgery, Heinrich-Heine-University Medical School, Duesseldorf, Germany;

[3] Institute for Medical Information Processing, Biometry and Epidemiology (IBE), Faculty of Medicine, LMU Munich, Munich, Germany)

E-Mail of corresponding author: christoph.olivier@uniklinik-freiburg.de

Supplement 1. Search strategy

PubMed:

("dialysis" OR "kidney failure, chronic"[Mesh]) AND "atrial fibrillation" AND (‘’Anticoagulant’’[Mesh] OR ‘’vitamin k antagonist’’ OR "Factor Xa Inhibitors"[Mesh] OR "apixaban" OR "rivaroxaban"[Mesh] OR "edoxaban" OR "dabigatran"[Mesh] OR "warfarin"[Mesh] OR "phenprocoumon"[Mesh])

Embase:

("chronic renal failure"/exp OR dialysis) AND "atrial fibrillation"/exp AND ("anticoagulation"/exp OR "vitamin k antagonist"/exp OR "direct oral anticoagulant"/exp)

Cochrane & ClinicalTrial.gov:

("chronic renal failure" OR "dialysis") AND ‘’Atrial fibrillation’’ AND (‘’Anticoagulation’’ OR ‘’ Vitamin k antagonist’’ OR ‘’direct oral anticoagulant’’)

Supplement 2. Bias assessment RCTs (RoB 2)

Study	De Vriese et al.	Pokorney et al.	Reinecke et al.	Harel et al.
Risk of bias arising from the randomization process	low	low	low	low
Risk of bias due to deviations from the intended interventions	low	low	low	low
Missing outcome data	low	low	low	low
Risk of bias in measurement of the outcome	low	low	low	low
Risk of bias in selection of the reported result	low	low	low	low
Overall risk of bias	**low**	**low**	**low**	**low**

Supplement 3. Bias assessment cohort studies (ROBINS-I V2)

Study	Chan et al.	Siontis et al.	See et al.	Wetmore et al.	Moore et al.	Laville et al.	Roh et al.
Bias due to confounding	low	low	low	low	low	low	low
Bias in classification of interventions	low	low	low	low	low	low	low
Bias in selection of participants into the study (or into the analysis)	low	low	low	low	low	low	low
Bias due to deviations from intended interventions	low	low	low	low	low	low	low
Bias due to missing data	low	low	low	low	low	low	low
Bias in measurement of the outcome	low	low	low	low	low	low	low
Bias in selection of the reported result	moderate	low	low	low	moderate	low	low
Overall risk of bias	**moderate**	**low**	**low**	**low**	**moderate**	low	low

Supplement 4. Funnel plots of RCTs and Cohort Studies

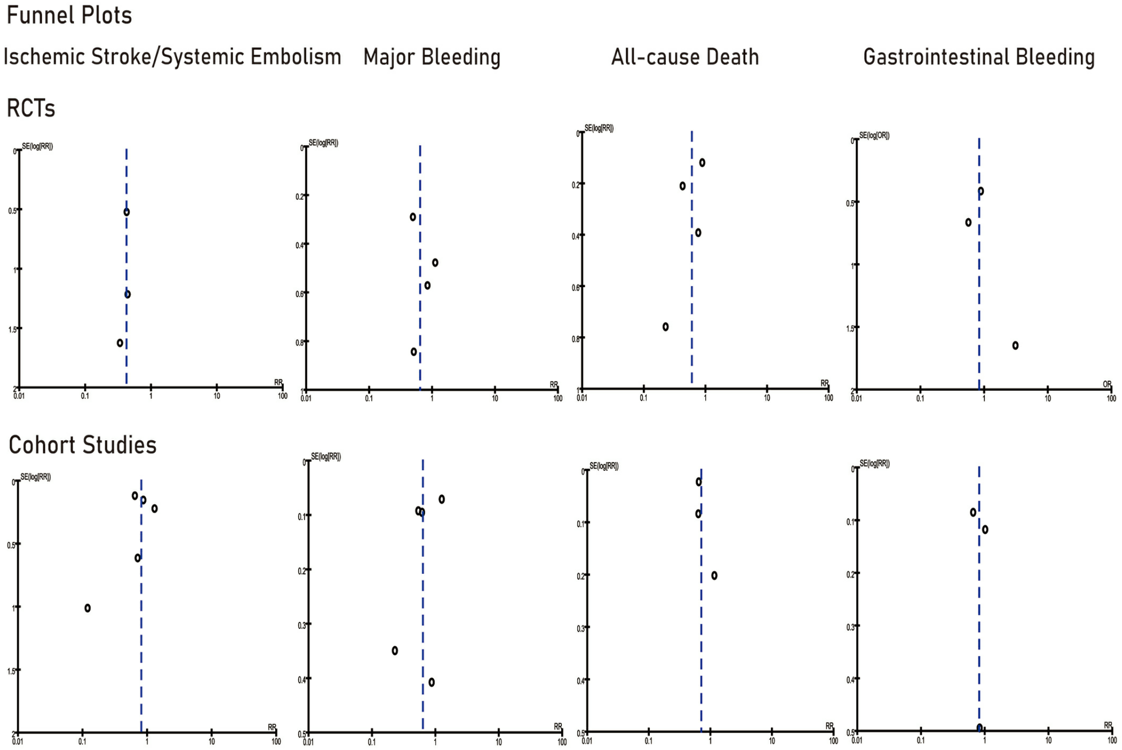

Supplement 5a. Forest plot of the composite outcome of ischemic stroke or systemic embolism, only cohort studies with low risk of bias included

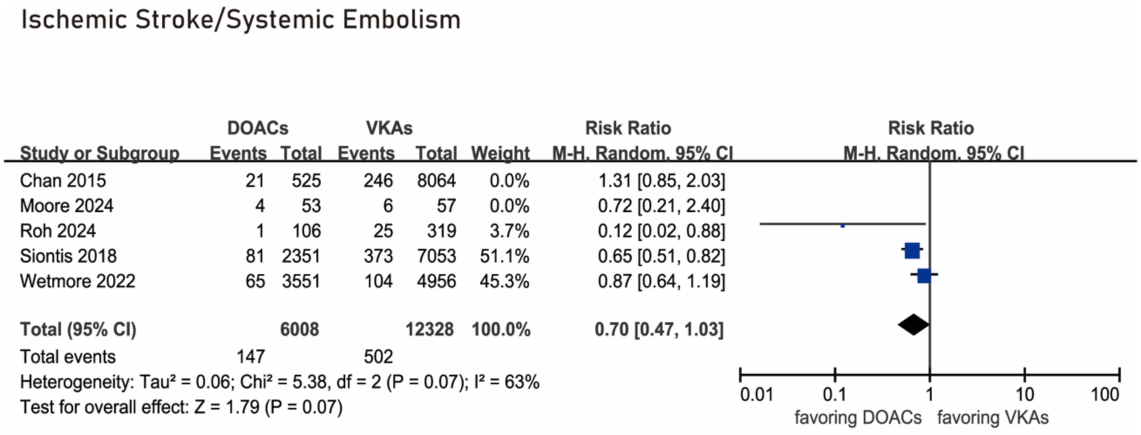

Study or Subgroup	DOACs Events	DOACs Total	VKAs Events	VKAs Total	Weight	Risk Ratio M-H, Random, 95% CI
Chan 2015	21	525	246	8064	0.0%	1.31 [0.85, 2.03]
Moore 2024	4	53	6	57	0.0%	0.72 [0.21, 2.40]
Roh 2024	1	106	25	319	3.7%	0.12 [0.02, 0.88]
Siontis 2018	81	2351	373	7053	51.1%	0.65 [0.51, 0.82]
Wetmore 2022	65	3551	104	4956	45.3%	0.87 [0.64, 1.19]
**Total (95% CI)**		**6008**		**12328**	**100.0%**	**0.70 [0.47, 1.03]**
Total events	147		502			

Heterogeneity: Tau² = 0.06; Chi² = 5.38, df = 2 (P = 0.07); I² = 63%
Test for overall effect: Z = 1.79 (P = 0.07)

Supplement 5b. Forest plot of major bleeding, only cohort studies with low risk of bias included

Major Bleeding

Study or Subgroup	DOACs Events	DOACs Total	VKAs Events	VKAs Total	Weight	Risk Ratio M-H, Random, 95% CI
Chan 2015	152	525	1858	8064	0.0%	1.26 [1.09, 1.45]
Laville 2024	8	184	437	2317	14.3%	0.23 [0.12, 0.46]
Moore 2024	9	53	11	57	0.0%	0.88 [0.40, 1.95]
Siontis 2018	129	2351	715	7053	43.1%	0.54 [0.45, 0.65]
Wetmore 2022	148	3551	334	4956	42.6%	0.62 [0.51, 0.75]
**Total (95% CI)**		**6086**		**14326**	**100.0%**	**0.51 [0.37, 0.69]**
Total events	285		1486			

Heterogeneity: Tau² = 0.05; Chi² = 7.86, df = 2 (P = 0.02); I² = 75%

Test for overall effect: Z = 4.37 (P < 0.0001)

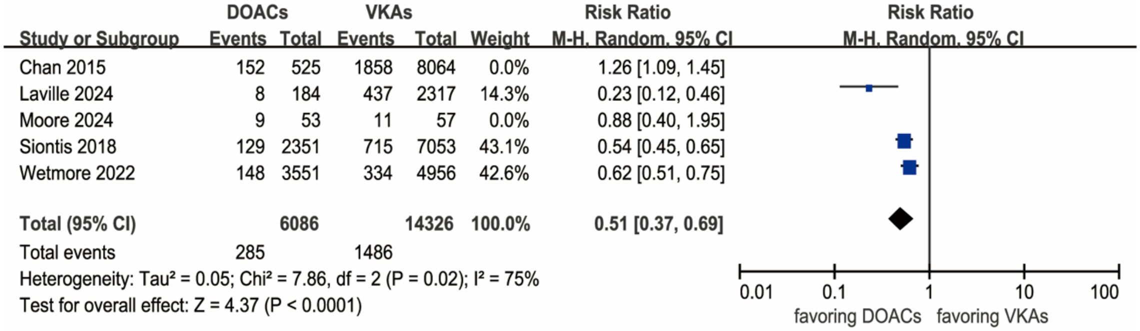

Supplement 5c. Forest plot of all-cause death, only cohort studies with low risk of bias included

All-cause Death

Study or Subgroup	DOACs Events	DOACs Total	VKAs Events	VKAs Total	Weight	Risk Ratio M-H, Random, 95% CI
Moore 2024	27	53	25	57	0.0%	1.16 [0.78, 1.72]
Siontis 2018	159	2351	753	7053	43.9%	0.63 [0.54, 0.75]
Wetmore 2022	877	3551	1559	4956	56.1%	0.79 [0.73, 0.84]
**Total (95% CI)**		**5902**		**12009**	**100.0%**	**0.71 [0.58, 0.88]**
Total events	1036		2312			

Heterogeneity: Tau² = 0.02; Chi² = 5.63, df = 1 (P = 0.02); I² = 82%

Test for overall effect: Z = 3.12 (P = 0.002)

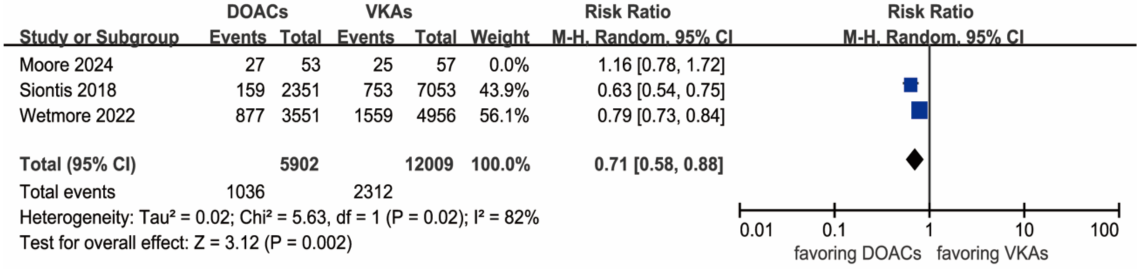

Supplement 5d. Forest plot of gastrointestinal bleeding, only cohort studies with low risk of bias included

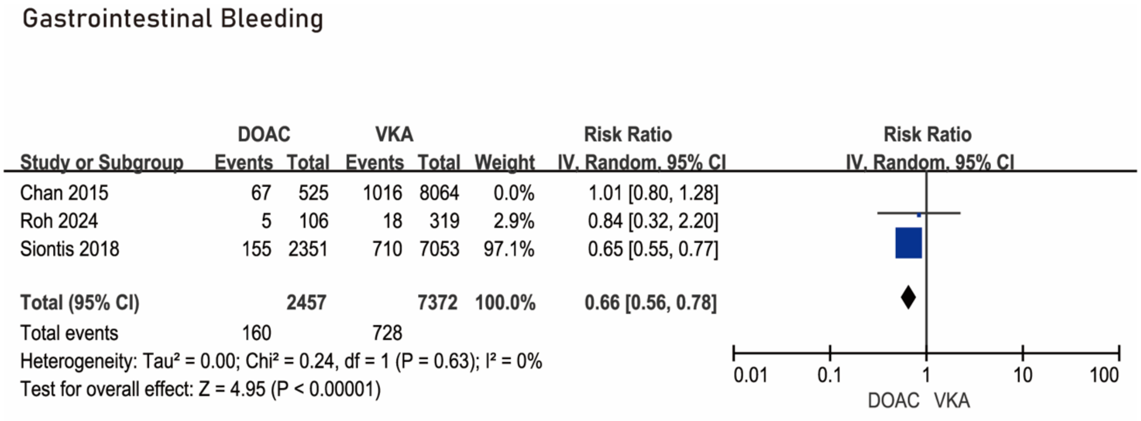

Supplement 6a. Forest plots of the composite outcome of ischemic stroke or systemic embolism, only studies investigating apixaban included

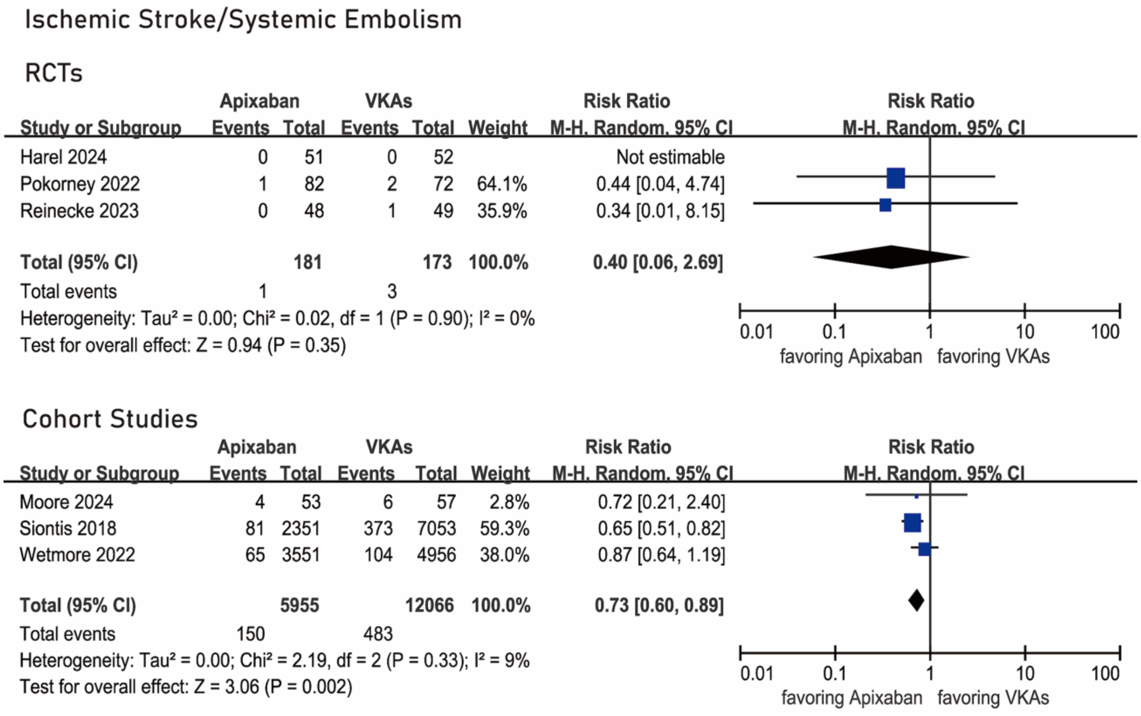

Supplement 6b. Forest plot of major bleeding, only studies investigating apixaban included

Major Bleeding

RCTs

Study or Subgroup	Apixaban Events	Apixaban Total	VKAs Events	VKAs Total	Weight	Risk Ratio M-H, Random, 95% CI
Harel 2024	2	51	4	52	15.9%	0.51 [0.10, 2.66]
Pokorney 2022	9	82	7	72	49.5%	1.13 [0.44, 2.88]
Reinecke 2023	5	48	6	49	34.7%	0.85 [0.28, 2.60]
**Total (95% CI)**		**181**		**173**	**100.0%**	**0.90 [0.47, 1.74]**
Total events	16		17			

Heterogeneity: Tau² = 0.00; Chi² = 0.69, df = 2 (P = 0.71); I² = 0%
Test for overall effect: Z = 0.31 (P = 0.76)

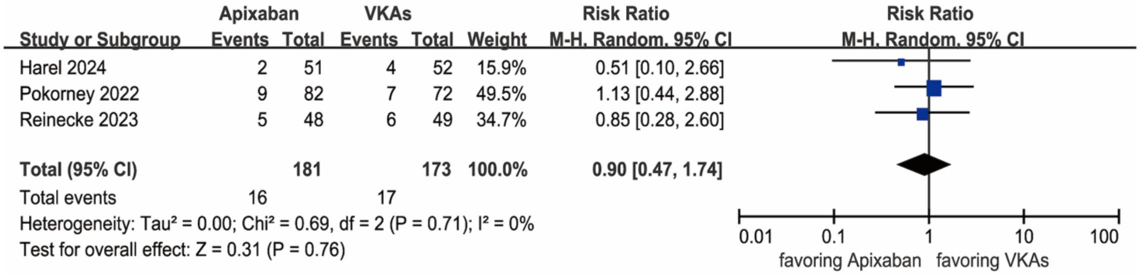

Cohort Studies

Study or Subgroup	Apixaban Events	Apixaban Total	VKAs Events	VKAs Total	Weight	Risk Ratio M-H, Random, 95% CI
Moore 2024	9	53	11	57	2.7%	0.88 [0.40, 1.95]
Siontis 2018	129	2351	715	7053	50.4%	0.54 [0.45, 0.65]
Wetmore 2022	148	3551	334	4956	46.9%	0.62 [0.51, 0.75]
**Total (95% CI)**		**5955**		**12066**	**100.0%**	**0.58 [0.51, 0.67]**
Total events	286		1060			

Heterogeneity: Tau² = 0.00; Chi² = 2.05, df = 2 (P = 0.36); I² = 2%
Test for overall effect: Z = 7.99 (P < 0.00001)

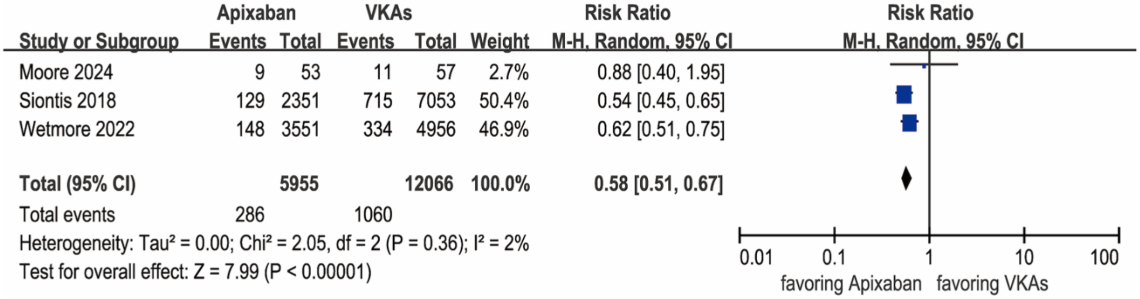

Supplement 6c. Forest plot of all-cause death, only studies investigating apixaban included

All-cause Death

RCTs

Study or Subgroup	Apixaban Events	Apixaban Total	VKAs Events	VKAs Total	Weight	Risk Ratio M-H, Random, 95% CI
Harel 2024	2	51	9	52	9.6%	0.23 [0.05, 1.00]
Pokorney 2022	21	82	43	72	61.4%	0.43 [0.28, 0.65]
Reinecke 2023	9	48	12	49	29.1%	0.77 [0.36, 1.65]
**Total (95% CI)**		**181**		**173**	**100.0%**	**0.48 [0.30, 0.77]**
Total events	32		64			

Heterogeneity: Tau² = 0.05; Chi² = 2.68, df = 2 (P = 0.26); I² = 25%
Test for overall effect: Z = 3.02 (P = 0.003)

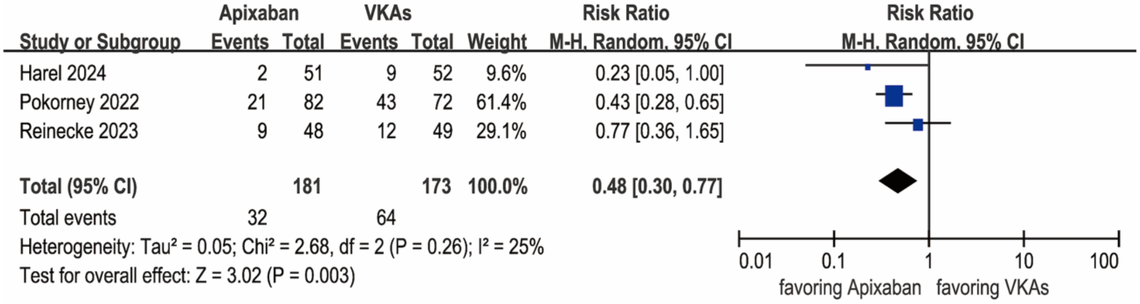

Cohort Studies

Study or Subgroup	Apixaban Events	Apixaban Total	VKAs Events	VKAs Total	Weight	Risk Ratio M-H, Random, 95% CI
Moore 2024	27	53	25	57	19.0%	1.16 [0.78, 1.72]
Siontis 2018	159	2351	753	7053	36.9%	0.63 [0.54, 0.75]
Wetmore 2022	877	3551	1559	4956	44.0%	0.79 [0.73, 0.84]
**Total (95% CI)**		**5955**		**12066**	**100.0%**	**0.78 [0.62, 0.98]**
Total events	1063		2337			

Heterogeneity: Tau² = 0.03; Chi² = 10.04, df = 2 (P = 0.007); I² = 80%
Test for overall effect: Z = 2.15 (P = 0.03)

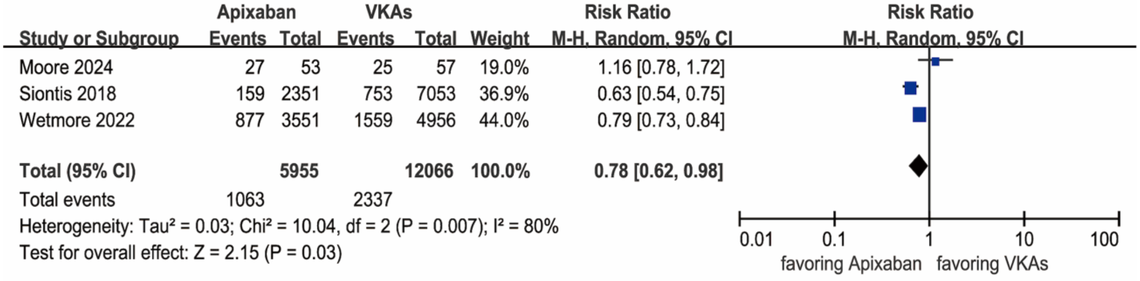

Supplement 7. Definitions of Major Bleeding

Study	Definition of Major Bleeding
De Vriese et al.	• A requirement for transfusion of two or more units of blood or<br>• A decrease in hemoglobin of 2 g/dl **and**<br>• Not fulfilling the criteria for life-threatening bleeding (Life-threatening bleeding: fatal bleeding; symptomatic intracranial bleeding; a decrease in hemoglobin of ≥ 5 g/dl; or a requirement for transfusion of four or more units of blood, inotropic agents , or surgery.)
Pokorney et al.	According to ISTH:<br>• Fatal bleeding, and/or<br>• Symptomatic bleeding in a critical area or organ, such as intracranial, intraspinal, intraocular, retroperitoneal, intra-articular or pericardial, or intramuscular with compartment syndrome, and/or<br>• Bleeding causing a fall in hemoglobin level of 20 g/L (1.24 mmol/L) or more, or leading to transfusion of two or more units whole blood or red cell.
Reinecke et al.	According to ISTH
Harel et al.	According to ISTH
Chan et al.	A hemorrhagic event resulting in hospitalization or death
Siontis et al.	When it was associated with a critical site code (such as intracranial), need for blood product transfusion based on a procedure code during the same admission, or death
See et al.	The total events of intracranial hemorrhage (defined using codes for atraumatic hemorrhage), major GIB (defined as a hospitalized primary code indicating bleeding in the gastrointestinal tract), and other critical site bleedings that required hospitalization
Wetmore et al.	• Fatal bleeding, and/or<br>• Involved a critical site, and/or<br>• Required a blood transfusion
Moore et al.	According to ISTH
Laville et al.	Bleeding that required hospitalization

Supplement 8. DOAC types and dosages

DOACs	Studies	Dosages and outcome data
Apixaban	Pokorney et al. (RCT)	Label-concordant (5 mg twice daily, or 2.5 mg twice daily if meeting at least 2 of the following criteria: creatinine ≥ 1.5 mg/dL, age ≥ 80 years, or body weight ≤ 60 kg)
	Reinecke et al. (RCT)	2.5 mg twice daily
	Harel et al. (RCT)	Label-concordant, but permitting the clinicians to reduce at their discretion.
	Siontis et al. (cohort)	2 subgroups receiving 5 mg twice daily or 2.5 mg twice daily regardless of indicated dosage. Separate outcome data for each subgroup available.
	Wetmore et al. (cohort)	2 subgroups receiving 5 mg twice daily as indicated or 2.5 mg twice daily although 5 mg twice daily indicated. Separate outcome data for each subgroup available.
	Moore et al. (cohort)	39.6% of patients received 5 mg twice daily, 60.4% received 2.5 mg twice daily, although 38.7% of them should have received 5 mg twice daily. No separate outcome data available.
Rivaroxaban	De Vriese et al. (RCT)	2 subgroups receiving either rivaroxaban 10 mg or rivaroxaban 10 mg and vitamin k2 supplement. Separate outcome data available.
	Chan et al. (cohort)	32,1% of patients received 20 mg per day and 67,8% received 15 mg per day. No separate outcome data available, only major bleeding was compared between the two subgroups.
Dabigatran	Chan et al. (cohort)	15.3% of patients received 150 mg twice daily, 84.7% received 75 mg twice daily. No separate outcome data available, only major bleeding was compared between the two subgroups.
Mixed	Lavielle et al. (cohort)	No information about the types or dosage of DOACs available for the subpopulation of patients with atrial fibrillation.
	Roh et al. (cohort)	No separate dosage information for each type of DOACs available, no separate outcome data for different DOAC types or dosages available.
